# Clinical Outcomes of Autologous Hematopoietic Stem Cell Transplantation in Filipino Patients With Multiple Sclerosis: A Single-Center Retrospective Case Series

**DOI:** 10.7759/cureus.102706

**Published:** 2026-01-31

**Authors:** Jon Stewart H Dy, Ludwig F Damian, Cymbeline Perez-Santiago, Francisco Lopez

**Affiliations:** 1 Neurosciences, St. Luke's Medical Center College of Medicine - William H. Quasha Memorial, Quezon City, PHL; 2 Neurology, St. Luke's Medical Center, Quezon City, PHL; 3 Neurology, Institute for Neurosciences, St. Luke's Medical Center, Quezon City, PHL; 4 Neurology, Institute for Neurosciences, St. Luke's Medical Center, Taguig City, PHL; 5 Bone Marrow and Transplant Service, St. Luke's Medical Center, Taguig City, PHL

**Keywords:** autologous hematopoietic stem cell transplantation, disease-modifying therapies, filipino patients, multiple sclerosis, no evidence of disease activity

## Abstract

Background: Autologous hematopoietic stem cell transplantation (aHSCT) is an emerging treatment option for patients with relapsing-remitting multiple sclerosis (RRMS) or secondary progressive multiple sclerosis (SPMS) who exhibit ongoing disease activity despite disease-modifying therapy (DMT). Limited access to high-efficacy DMTs in the Philippines makes aHSCT an important escalation strategy. This study describes the clinical characteristics, transplant course, and short-term outcomes of Filipino patients with multiple sclerosis (MS) who underwent aHSCT at a single tertiary center.

Methodology: We conducted a retrospective observational review of all patients with MS who underwent aHSCT as salvage or escalation therapy for ongoing disease activity despite prior DMTs at St. Luke's Medical Center from April 2023 to March 2025. Extracted data included demographics, MS phenotype, disease duration, baseline neurologic functional status, mobilization and conditioning regimens, transplant-related complications, magnetic resonance imaging (MRI) findings, post-transplant clinical outcomes, and No Evidence of Disease Activity (NEDA) status. Conditioning regimens included carmustine, cytarabine, etoposide, melphalan, and anti-thymocyte globulin (ATG) in five patients (83.3%) and cyclophosphamide-methylprednisolone-ATG in one patient (16.7%). Descriptive statistics were used to summarize outcomes.

Results: Six Filipino patients (median age: 44.5 years) were included. Five patients (83.3%) were female, and one (16.7%) was male. Five patients (83.3%) had SPMS, and one patient (16.7%) had RRMS. The mean disease duration was 14.3 years, and five patients (83.3%) had moderate to severe neurologic disability at baseline requiring ambulatory assistance. Rituximab was the most commonly used pre-aHSCT DMT. Febrile neutropenia occurred in five patients (83.3%), and one patient (16.7%) developed a central line-associated bloodstream infection; all patients recovered. During a mean follow-up of 17.2 months, no patient experienced clinical relapse or worsening functional disability, and none required resumption of DMTs. Two patients (33.3%) who underwent interval MRI demonstrated no new T2 or gadolinium-enhancing lesions. Four patients (66.7%) achieved NEDA-2, and two patients (33.3%) with interval MRI follow-up achieved NEDA-3.

Conclusions: aHSCT was well tolerated and associated with short-term clinical stability in Filipino patients with active RRMS and SPMS. During follow-up, all patients remained relapse-free without evidence of confirmed disability progression. These findings support the role of aHSCT as a viable therapeutic option in settings with limited access to high-efficacy DMTs. Larger studies with longer follow-up are needed to evaluate the durability of remission.

## Introduction

Multiple sclerosis (MS) is the most common demyelinating central nervous system disorder [1,2]. Disease-modifying therapies (DMTs) remain the cornerstone of treatment for relapsing-remitting MS (RRMS) and secondary progressive MS (SPMS) [3]. However, in the Philippines, access to high-efficacy DMTs is limited, with only beta-interferon, fingolimod, rituximab, ocrelizumab, and autologous hematopoietic stem cell transplantation (aHSCT) currently available [4]. For patients with ongoing disease activity or inadequate treatment response despite DMTs, aHSCT represents a potential escalation strategy aimed at immune reconstitution and durable disease control.

We previously reported the first Filipino patient with RRMS who successfully underwent aHSCT, highlighting its feasibility in a local setting [5]. Building on that experience, the objective of this study was to describe the clinical outcomes, short-term neurologic course, and transplant-related safety profile of six Filipino patients with active RRMS and SPMS who underwent aHSCT at our institution. To our knowledge, this is the first local case series documenting outcomes of aHSCT for MS in the Philippines. This report underscores the potential role of aHSCT as a therapeutic option within specialized centers, particularly in resource-limited settings where access to high-efficacy DMTs remains constrained.

## Materials and methods

Study design and setting

This was a retrospective, single-center observational study that included all Filipino patients with MS who underwent aHSCT at St. Luke’s Medical Center, Philippines, between April 2023 and March 2025. Clinical data were extracted from electronic medical records.

Patient selection

Patients were included if they had a confirmed diagnosis of RRMS or SPMS, evidence of ongoing disease activity despite treatment with at least one DMT, and underwent aHSCT at our institution during the study period. Eligible patients were required to have available pre-transplant clinical data and post-transplant follow-up information.

Patients were excluded if they had an alternative neurologic diagnosis that could account for neurologic deficits, incomplete clinical documentation, or insufficient follow-up data to assess post-transplant outcomes. Medical comorbidities were not exclusionary unless they confounded neurologic assessment or outcome interpretation.

Mobilization and conditioning regimens

All patients underwent stem cell mobilization using granulocyte colony-stimulating factor (G-CSF) at a dose of 10 mcg/kg/day for five days. No concurrent chemotherapy was administered during mobilization. The interval between mobilization and autologous stem cell infusion ranged from 7 to 14 days.

Two conditioning protocols were used based on clinical assessment and institutional discretion. Five patients (83.3%) received a conditioning regimen consisting of carmustine, cytarabine, etoposide, melphalan, and anti-thymocyte globulin (ATG) derived from horse serum. One patient (16.7%) received an alternative conditioning regimen consisting of cyclophosphamide, methylprednisolone, and ATG derived from rabbit serum. Selection of the conditioning regimen was individualized and determined through multidisciplinary discussion, taking into account disease severity, prior DMT exposure, comorbidities, and transplant-related risk assessment.

Following conditioning, autologous CD34-positive hematopoietic stem cells were reinfused on Day 0. All patients received institutional standard supportive care, including antimicrobial prophylaxis and close monitoring for transplant-related complications.

Data collection

Extracted data included demographics (age and sex), MS phenotype, duration of illness, baseline neurologic functional status, pre-aHSCT treatments (including prior DMT exposure), pre-transplant magnetic resonance imaging (MRI) findings, details of mobilization and conditioning regimens, transplant-related complications (including infectious and hematologic adverse events), post-aHSCT clinical outcomes (including relapses and functional disability status), MRI activity during follow-up, duration of follow-up, post-transplant DMT use, and achievement of No Evidence of Disease Activity (NEDA) status.

Post-transplant MRI follow-up was performed based on clinical indication and imaging availability rather than a standardized surveillance protocol, reflecting real-world retrospective practice at our institution.

Neurologic disability and functional outcomes were assessed based on routine clinical neurologic examinations and documentation of ambulatory status, need for assistance or assistive devices, and activities of daily living as recorded in the medical records. Functional stability was defined as the absence of clinical relapses and no documented worsening of baseline ambulatory or functional status during follow-up.

Ethics statement

This study involved a retrospective review of fully de-identified patient data. The study was reviewed and registered by the St. Luke’s Medical Center Institutional Scientific Review Committee and Institutional Ethics Review Committee and was formally granted exemption from ethical review in accordance with institutional guidelines.

Statistical analysis

Given the small sample size, all analyses were descriptive. Continuous variables are presented as means and ranges, while categorical variables are summarized as frequencies and percentages. No inferential statistical testing was performed.

## Results

Baseline characteristics

**Figure 1:**
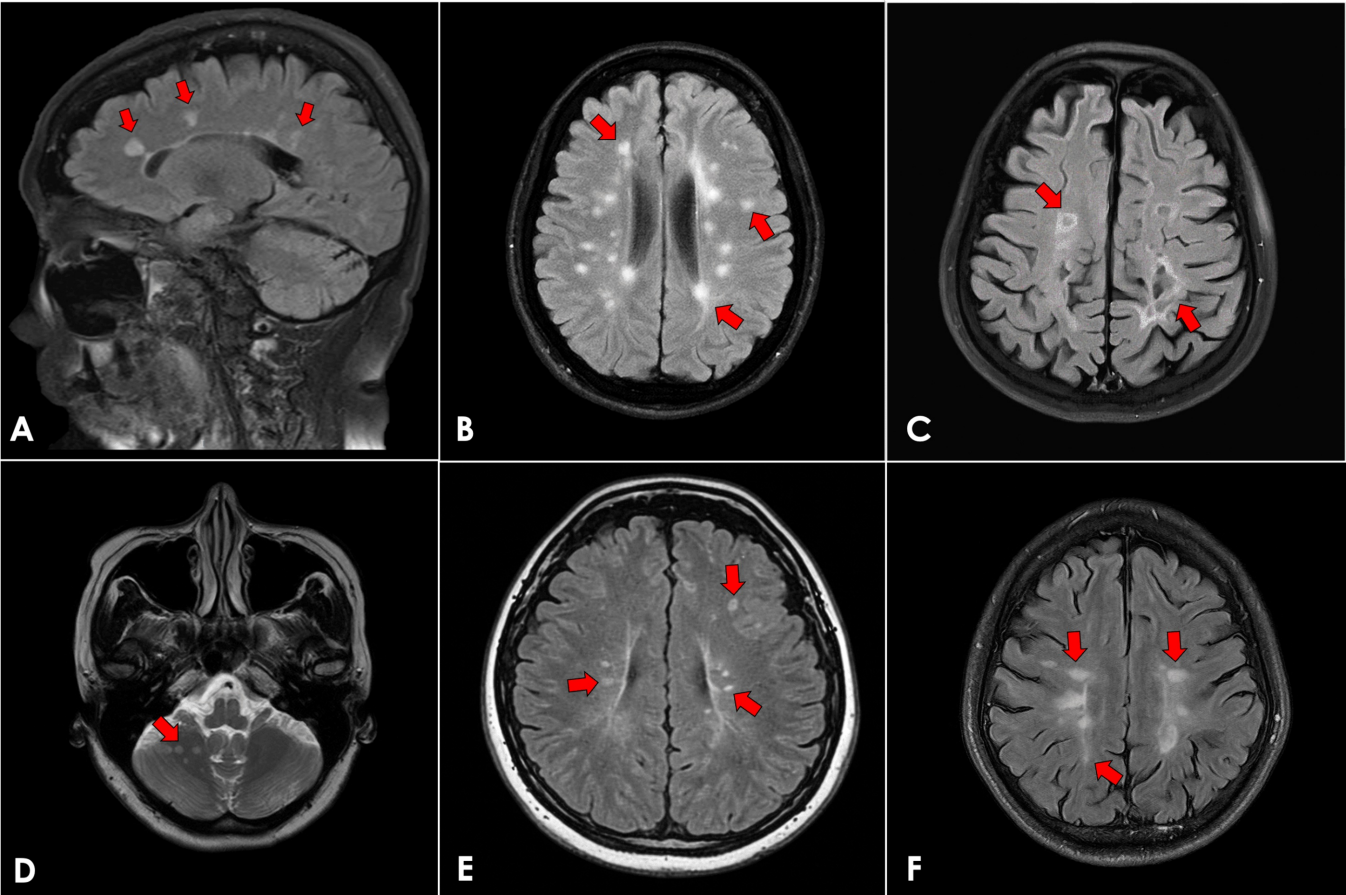
Representative pre-aHSCT brain MRI images. (A) Case 1: sagittal FLAIR image demonstrating periventricular hyperintense lesions (arrows). (B) Case 2: axial FLAIR image showing hyperintense lesions in the periventricular, juxtacortical, and subcortical regions (arrows). (C) Case 3: axial FLAIR image demonstrating periventricular and subcortical hyperintense lesions (arrows). (D) Case 4: axial T2-weighted image showing hyperintense lesions in the cerebellar hemisphere (arrow). (E) Case 5: axial FLAIR image demonstrating juxtacortical and periventricular hyperintense lesions (arrows). (F) Case 6: axial FLAIR image showing juxtacortical and subcortical hyperintense lesions (arrows) Abbreviations: aHSCT = autologous hematopoietic stem cell transplantation; MRI = magnetic resonance imaging; FLAIR = fluid-attenuated inversion recovery

Six Filipino patients with MS underwent aHSCT during the study period. The median age was 44.5 years (range: 33-56 years). Five patients (83.3%) were female, and one patient (16.7%) was male. Five patients (83.3%) had SPMS, and one patient (16.7%) had RRMS. The mean disease duration was 14.3 years (range: 8-25 years). At baseline, five patients (83.3%) had moderate to severe neurologic disability requiring ambulatory assistance, a clinical profile commonly seen in patients with advanced or treatment-refractory multiple sclerosis and frequently encountered among candidates for aHSCT. Rituximab was the most commonly used pre-aHSCT DMT (n = 5, 83.3%). The interval between stem cell mobilization and aHSCT ranged from 7 to 14 days across all patients, in accordance with institutional protocol. Baseline demographic and clinical characteristics are summarized in Table 1. Representative pre-aHSCT MRI findings are shown in Figure 1.

**Table 1:** The patients’ representative profiles pre-aHSCT Abbreviations: DMT = disease-modifying therapy; aHSCT = autologous hematopoietic stem cell transplantation; RRMS = relapsing–remitting MS; SPMS = secondary progressive MS; y.o. = years old; ADLs = activities of daily living

Case	Age (Years)	Sex	MS Phenotype	Disease Duration (Years)	Baseline Functional Status	Pre-aHSCT DMT(s)
1	56 y.o.	Female	RRMS	25	Fully ambulatory, minimal functional impairment	Rituximab every 6 months for 2 years
2	44 y.o.	Female	SPMS	14	Ambulatory with assistance	Fingolimod every other day for 4 years and rituximab every 6 months for 5 years
3	44 y.o.	Female	SPMS	22	Ambulatory with assistance	Methotrexate every week for 4 years
4	41 y.o.	Female	SPMS	8	Ambulatory with assistance; requires help with some ADLs	Rituximab every 6 months for 4 years and azathioprine daily for 1 year
5	45 y.o.	Female	SPMS	18	Significant gait impairment requiring assistance	Beta-interferon thrice weekly and rituximab every 6 months for 2 years
6	33 y.o.	Male	SPMS	9	Ambulatory with an assistive device	Rituximab every 6 months for 6 years

Individual case summaries

Case 1

A 56-year-old woman with a 25-year history of RRMS experienced recurrent relapses despite treatment with rituximab and developed new-onset first-degree atrioventricular block. At baseline, she was fully ambulatory with minimal functional impairment. Pre-aHSCT MRI demonstrated stable multifocal T2-fluid-attenuated inversion recovery (FLAIR) hyperintense lesions involving the brain and cervical and thoracic spinal cord. She received conditioning with carmustine, cytarabine, etoposide, melphalan, and horse-derived ATG. Apart from febrile neutropenia, the post-transplant course was uneventful. At 30-month follow-up, she remained relapse-free, with no evidence of new neurologic deficits, stabilization of pre-existing neurologic function compared with baseline, and no new MRI lesions.

Case 2

A 44-year-old woman with RRMS evolving to SPMS over 14 years had persistent relapses despite fingolimod and rituximab. At baseline, she required ambulatory assistance due to lower extremity weakness. Pre-aHSCT MRI revealed stable T2-FLAIR hyperintense lesions involving the brainstem and cerebellum. She underwent conditioning with carmustine, cytarabine, etoposide, melphalan, and horse ATG. Febrile neutropenia occurred but resolved with antibiotic therapy. At 25-month follow-up, she remained relapse-free, with no evidence of new neurologic deficits and stabilization of pre-existing neurologic function compared with baseline.

Case 3

A 44-year-old woman with RRMS evolving to SPMS over 22 years experienced recurrent relapses while on methotrexate. Baseline functional status was characterized by moderate neurologic disability requiring ambulatory assistance. Pre-aHSCT MRI demonstrated multifocal supratentorial T2-FLAIR hyperintense lesions. She received conditioning with carmustine, cytarabine, etoposide, melphalan, and horse ATG. The post-transplant course was unremarkable. At the 23-month follow-up, she remained relapse-free, with no evidence of new neurologic deficits and stabilization of pre-existing functional impairment compared with baseline, and no new MRI lesions.

Case 4

A 41-year-old woman with RRMS progressing to SPMS over eight years had multiple relapses despite treatment with rituximab and azathioprine. At baseline, she required ambulatory assistance and support for some activities of daily living. Pre-aHSCT MRI showed T2-FLAIR hyperintense lesions involving the periventricular region, pons, and cerebellum. She underwent conditioning with carmustine, cytarabine, etoposide, melphalan, and horse ATG. Her post-transplant course was complicated by febrile neutropenia and transient pulmonary congestion, both of which resolved with appropriate management. At the 21-month follow-up, she remained relapse-free, with no evidence of new neurologic deficits and stabilization of pre-existing functional limitations compared with baseline.

Case 5

A 45-year-old woman with RRMS evolving to SPMS over 18 years experienced persistent relapses despite beta-interferon and rituximab therapy. Baseline neurologic disability included significant gait impairment requiring ambulatory assistance. Comorbidities included stage I invasive ductal carcinoma, chronic deep venous thrombosis, and dyslipidemia. Pre-aHSCT MRI demonstrated multifocal T2-FLAIR hyperintense lesions involving the brain and thoracic spinal cord. She received conditioning with carmustine, cytarabine, etoposide, melphalan, and horse ATG. Her post-transplant course was complicated by a central line-associated bloodstream infection, which was successfully treated with antibiotics. At the 18-month follow-up, she remained relapse-free, with no evidence of new neurologic deficits and stabilization of pre-existing functional impairment compared with baseline.

Case 6

A 33-year-old man with RRMS progressing to SPMS over nine years experienced relapses despite rituximab therapy. At baseline, he had moderate neurologic disability with gait impairment requiring the use of an assistive device. Pre-aHSCT MRI revealed T2-FLAIR hyperintense lesions in the juxtacortical, subcortical, periventricular, and cerebellar regions. He received conditioning with cyclophosphamide, methylprednisolone, and rabbit-derived ATG. His post-transplant course was complicated by febrile neutropenia, which resolved with antibiotic treatment. At the seven-month follow-up, he remained relapse-free, with no evidence of new neurologic deficits and stabilization of pre-existing functional impairment compared with baseline.

Post-transplant clinical and MRI outcomes

Across the cohort, the mean follow-up duration was 17.2 months (range: 3-24 months). No patient experienced a clinical relapse during follow-up, and no patient demonstrated worsening functional neurologic disability. No patient required resumption of DMTs after aHSCT. Two patients (33.3%) underwent interval follow-up brain MRI, which demonstrated no new or enlarging T2-FLAIR lesions and no gadolinium-enhancing lesions, corresponding to NEDA with radiologic confirmation (NEDA-3). The remaining four patients (66.7%) achieved NEDA based on clinical stability alone (NEDA-2). Post-transplant outcomes are summarized in Table 2.

**Table 2:** The patients' representative profiles post-HSCT Abbreviations: aHSCT = autologous hematopoietic stem cell transplantation; MRI = magnetic resonance imaging; DMT = disease-modifying therapy; NEDA = no evidence of disease activity

Case	Major Transplant-Related Complications	Follow-Up Duration (Months)	Post-aHSCT Clinical Relapses	Post-aHSCT MRI Relapses	Functional Disability Progression	DMT (Post-aHSCT)	NEDA Status
1	Febrile neutropenia	30	None	No new lesions	None	None	3
2	Febrile neutropenia	25	None	Not performed	None	None	2
3	None	23	None	No new lesions	None	None	3
4	Febrile neutropenia, transient pulmonary congestion	21	None	Not performed	None	None	2
5	Central line-associated bloodstream infection	18	None	Not performed	None	None	2
6	Febrile neutropenia	7	None	Not performed	None	None	2

## Discussion

MS is a leading cause of disability in young adults [1], and treatment decisions are shaped by disease activity, comorbidities, and access to high-efficacy DMTs [3,6,7]. In the Philippines, limited availability of DMTs and real-world barriers to treatment often complicate long-term disease management. This case series highlights Filipino patients with active RRMS or SPMS who demonstrated suboptimal response to available DMTs and subsequently underwent aHSCT.

Rituximab was the most commonly used high-efficacy DMT among our patients. Although widely utilized in local practice [8], rituximab remains off-label for MS [7,8]. Consistent with global experience, many of our patients continued to exhibit relapses or disability progression despite anti-CD20 therapy. In settings where a limited number of high-efficacy DMTs are accessible, therapeutic escalation options become restricted, making aHSCT a viable consideration for patients with ongoing inflammatory activity [8].

International guidelines describe ideal candidates for aHSCT as those younger than 50 years old, with relatively early disease duration, preserved ambulation, and persistent disease activity despite treatment [8]. Our cohort largely met these criteria, although many had longstanding disease and had already transitioned to SPMS. The conditioning regimens were tolerated, and expected toxicities, most commonly febrile neutropenia, were successfully managed. No serious complications or transplant-related mortality occurred, similar to reported rates of 0.2-2% in published literature [8,9]. All patients were hospitalized for approximately 3.5 weeks, aligned with standard transplant protocols, and no patient required resumption of DMT post-aHSCT [9].

Evidence increasingly supports the efficacy of aHSCT in suppressing inflammatory disease activity, reducing relapse rates, and stabilizing disability [9,10]. International studies report NEDA rates of 70-92%, with durable remission lasting up to 15 years in many cohorts [11]. Disability improvement, particularly among patients with active SPMS, has been documented in approximately one-third of patients at three-year follow-up [10-13]. All our patients achieved clinical remission during short-term follow-up, with no relapses, no new MRI activity in those who underwent imaging, and stabilization of neurologic function. These outcomes are consistent with published data, although longer follow-up is needed to determine whether these effects are durable.

Our findings have clinical implications for MS management in resource-limited settings, where treatment decisions depend not only on disease characteristics but also on medication availability, cost, and access to specialized centers with expertise in the multidisciplinary management of advanced MS and aHSCT. Notably, most of our patients progressed to SPMS before undergoing aHSCT, suggesting that earlier therapeutic escalation, prior to substantial disability accumulation, may yield more favorable outcomes [11,14]. Based on our experience, Filipino patients with highly active RRMS who fail at least one high-efficacy DMT may benefit from earlier consideration of aHSCT.

This study has several limitations. The small sample size and retrospective, single-center design limit generalizability, and all patients were of the same ethnic background. Neurologic disability outcomes were assessed using descriptive clinical measures derived from routine neurologic examinations, such as ambulatory status, use of assistive devices, and activities of daily living, rather than standardized numeric disability scales, reflecting real-world practice in our setting. Follow-up duration was relatively short, and post-transplant MRI surveillance was not standardized, with imaging obtained based on clinical indication and availability. The absence of a control group receiving alternative high-efficacy DMTs limits comparative assessment of treatment effectiveness. Additionally, heterogeneity in conditioning regimens reflects individualized clinical decision-making rather than a uniform protocol. Access to aHSCT remains limited to select tertiary centers, and standardized referral pathways for MS are lacking. Larger, multicenter studies with longer follow-up, standardized outcome measures, and comparative cohorts are needed to better define the long-term efficacy and safety of aHSCT in Filipino patients with MS.

## Conclusions

In summary, this case series represents the first local report describing outcomes of Filipino patients with MS who underwent aHSCT. All patients demonstrated short-term clinical stability, supporting the potential role of aHSCT as a therapeutic option for individuals with active disease and inadequate response to available disease-modifying therapies. These findings highlight the importance of considering aHSCT earlier in the disease course, before the accumulation of substantial and irreversible disability. Larger studies incorporating standardized treatment protocols, longer follow-up, and direct comparisons with other high-efficacy DMTs are needed to better define the long-term safety, efficacy, and cost-effectiveness of aHSCT in Filipino patients with MS.
